# Prevalence of obesity and cardiovascular risk among children and adolescents in the municipality of Santa Cruz do Sul, Rio Grande do Sul

**DOI:** 10.1590/1516-3180.2013.1315518

**Published:** 2013-10-01

**Authors:** Cézane Priscila Reuter, Leandro Tibiriçá Burgos, Marcelo Dias Camargo, Lia Gonçalves Possuelo, Miriam Beatris Reckziegel, Éboni Marília Reuter, Francielle Pasqualotti Meinhardt, Miria Suzana Burgos

**Affiliations:** I BSc. Pharmacist. Master Degree in Health Promotion, Universidade de Santa Cruz do Sul (UNISC), Santa Cruz do Sul.; Doctoral studentin Human Movement Sciences, Universidade Federal do Rio Grande do Sul (UFRGS), Porto Alegre, Rio Grande do Sul, Brazil.; II MSc. Professor, Department of Physical Education and Health, Universidade de Santa Cruz do Sul (UNISC), Santa Cruz do Sul, Rio Grande do Sul, Brazil.; III MSc. Doctoral Student in Health Sciences, Cardiology and Cardiovascular Sciences, Universidade Federal do Rio Grande do Sul/Hospital de Clínicas de Porto Alegre (UFRGS/HCPA).; Physical Educator, Hospital de Clínicas de Porto Alegre (HCPA), Porto Alegre, Rio Grande do Sul, Brazil.; IV PhD. Professor, Department of Biology and Pharmacy and Postgraduate Course (Stricto Sensu) on Health Promotion, Universidade de Santa Cruz do Sul (UNISC), Santa Cruz do Sul, Rio Grande do Sul, Brazil.; V BSc. Physiotherapist. Master's Student in Health Promotion, Universidade de Santa Cruz do Sul (UNISC), Santa Cruz do Sul, Rio Grande do Sul, Brazil.; VI BSc. Pharmacist and Master's student in Health Promotion Universidade de Santa Cruz do Sul (UNISC), Santa Cruz do Sul, Rio Grande do Sul, Brazil.; VII PhD. Professor, Department of Physical Education and Health and Postgraduate Course (Stricto Sensu) on Health Promotion, University of Santa Cruz do Sul (UNISC), Santa Cruz do Sul, Rio Grande do Sul, Brazil.

**Keywords:** Obesity, Blood pressure, Lipids, Glucose, Epidemiology, Obesidade, Pressão arterial, Lipídeos, Glucose, Epidemiologia

## Abstract

**CONTEXT AND OBJECTIVE:**

Studies have demonstrated that metabolic complications from child obesity, although silent, increase the risk of development of cardiovascular diseases in adulthood. The present paper sought to describe the prevalence of overweight/obesity and analyze the possible relationship between obesity and other cardiovascular risk factors among children and adolescents.

**DESIGN AND SETTING:**

Cross-sectional study, conducted in a university.

**METHODS:**

The study included 564 children and adolescents, aged 8 to 17 years. Body mass index and waist circumference were used to evaluate obesity. Other cardiovascular risk factors were evaluated, like systolic and diastolic blood pressure, glycemia, triglycerides and total cholesterol. Descriptive analysis was used for sample characterization, the chi-square test for categorical variables and Pearson's linear correlation for evaluating the relationship between obesity indicators and other cardiovascular risk factors.

**RESULTS:**

High prevalence of overweight/obesity was found among the schoolchildren (25.3% among the boys and 25.6% among the girls), along with abdominal obesity (19.0%). The overweight/obese schoolchildren presented higher percentages for the pressure and biochemical indicators, compared with underweight and normal-weight schoolchildren. Body mass index and waist circumference showed a weak correlation with the variables of age and systolic and diastolic blood pressure (P < 0.001), but there was no correlation between these obesity indices and biochemical variables.

**CONCLUSION:**

The high prevalence of overweight/obesity and its relationship with other cardiovascular risk factors demonstrate that it is necessary to develop intervention and prevention strategies from childhood onwards, in order to avoid development of chronic-degenerative diseases in adulthood.

## Abstract (pt)

**CONTEXTO E OBJETIVO:**

Estudos demonstram que as complicações metabólicas da obesidade infantil, mesmo silenciosas, aumentam o risco de desenvolvimento de doenças cardiovasculares na fase adulta. O presente estudo buscou descrever a prevalência de sobrepeso/obesidade, analisando possível relação entre obesidade e demais fatores de risco cardiovascular em crianças e adolescentes.

**TIPO DE ESTUDO E LOCAL:**

Estudo transversal, realizado em universidade.

**MÉTODOS:**

Foram incluídas 564 crianças e adolescentes de 8 a 17 anos. Foi utilizado o índice de massa corporal e a circunferência da cintura para avaliar obesidade. Outros fatores de risco cardiovasculares foram avaliados, como pressão arterial sistólica e diastólica, glicemia, triglicerídeos e colesterol total. Foi utilizada a análise descritiva para caracterização da amostra, teste de qui-quadrado para variáveis categóricas e correlação linear de Pearson para avaliar a relação entre os indicadores de obesidade e demais fatores de risco cardiovasculares.

**RESULTADOS:**

Foi encontrada alta prevalência de sobrepeso/obesidade entre os escolares (25,3% entre os meninos e 25,6% entre as meninas), assim como obesidade abdominal (19,0%). Escolares com sobrepeso/obesidade apresentaram percentual superior para os indicadores bioquímicos e pressóricos, em comparação aos escolares com baixo peso/normal. Foi encontrada correlação fraca entre índice de massa corporal e circunferência da cintura com as variáveis: idade, pressão arterial sistólica e pressão arterial diastólica (P < 0,001), não havendo correlação entre esses índices de obesidade com as variáveis bioquímicas.

**CONCLUSÃO:**

A alta prevalência de sobrepeso/obesidade encontrada, bem como sua relação com demais fatores de risco cardiovasculares, demonstram que é necessário o desenvolvimento de estratégias de intervenção e prevenção desde a infância, evitando o aparecimento de doenças crônico-degenerativas na fase adulta.

## INTRODUCTION

Obesity, which is characterized by excessive accumulation of body fat,^1^ is associated with several comorbidities, like cardiovascular diseases, type II diabetes, high blood pressure, certain types of cancer and other health complications starting in childhood.^1-5^ It is one of the most serious public health problems today: obesity and overweight problems are continually rising among children and adolescents throughout Brazil.^6^

One of the mechanisms that correlate obesity with cardiovascular conditions is the fact that a huge number of bioactive mediators are released through adipose tissue. These not only exert an influence on homeostasis of body weight, but also on insulin resistance and alterations to lipid profile, blood pressure, coagulation, fibrillosis and inflammation, thus resulting in endothelial dysfunction and atherosclerosis.^7^ Moreover, cardiac complications like coronary heart disease, cardiac insufficiency and sudden death have been correlated with a variety of adaptations or alterations to the cardiac structure and function that take place when adipose tissue accumulates excessively.^3,5^

The role of genetics in the development of some pathological conditions is well known. It has been demonstrated that when this component is associated with stimuli produced during childhood, permanent adaptive responses that cause long-term changes to tissue structure or function ensue.^8^ Consequently, obesity in children and adolescents not only has short-term implications but also produces risk factors for development of conditions of obesity and resultant comorbidities in adulthood.^5^

On the other hand, excessive weight can be controlled more easily during childhood than can the behavior and lifestyles of the parents and other family members.^9^ Children need higher calorie consumption because they are in the growth stage, and also have the chance to spend more time on leisure activities than adults can.^10^ Evidence also suggests that early treatment is more effective than at older ages.^9^ Furthermore, children and adolescents who lead a healthy and active lifestyle become conscious adults, which may help in preventing diseases and promoting health and quality of life, thus increasing their life expectancy.^11^

Although there is no common opinion in the literature with regard to which interventions are more effective for reducing the levels of obesity, most approaches tend to be focused on lifestyle changes, comprising nutritional reeducation and stimulation of physical activity.^12^ Such changes have been shown to reduce the incidence of several chronic-degenerative diseases, with consequent reduction in cardiovascular-related deaths among obese individuals.^13^

To this end, it is extremely important to conduct studies that address the prevalence and dynamics of chronic conditions like obesity that are public health issues. In this manner, the real needs of the population can be addressed, and strategies focusing on guided interventions can be put forward, with the aim of avoiding chronic diseases.^14,15^

### OBJECTIVE

The aims of the present study were to describe the prevalence of overweight/obesity condition and analyze possible associations with cardiovascular risk factors like blood pressure and biochemical variables. 

### METHODS

### Selection of participants 

The subjects of this cross-sectional study were 564 children and adolescents (42.0% males and 58.0% females), aged 8 to 17, who were attending 15 municipal, state and private schools located in the town and countryside of Santa Cruz do Sul, Rio Grande do Sul, Brazil. 

The participants were selected randomly, based on stratified cluster sampling (center and northern, southern, eastern and western outskirts of the town area, and northern, southern, eastern and western parts of the rural zone). Therefore, initially, a survey was conducted on the numbers and names of all municipal, state and private schools across the municipality, as well as a survey on the total number of schoolchildren, at the 6th Regional Educational Office of the state of Rio Grande do Sul and at the Municipal Education Department of Santa Cruz do Sul. This showed that, in total, 20,380 students were regularly enrolled. 

The sampling was carried out at random (lots were drawn for selecting the schools), proportional to the number of students per region. To determine the minimum number of participants per sample, the Nea Research Division formula, cited by Christensen,^16^ was used. This formula suggested that, for a population of 20,380 children and adolescents, the sample should consist of approximately 392 participants, with a 5% error margin. 

Fourteen losses were registered (2.4%), comprising schoolchildren who did not belong to the age group selected for the study. Two age groups were defined: under-12-year-olds were classified as children; while individuals aged 12 to 18 were considered to be adolescents, in accordance with the Brazilian Child and Adolescent Statute.^17^

### Ethical considerations in the study 

The project was firstly forwarded to and approved by the Ethics Committee for Research on Human Beings of the University of Santa Cruz do Sul (Universidade de Santa Cruz do Sul, UNISC), under protocol number 1189/05 and official letter number 240/05, in compliance with the Declaration of Helsinki. The children's parents or guardians signed a free and informed consent statement, thereby authorizing their children to take part in the investigation of cardiovascular disease risk factors, authorizing their participation in the evaluations and tests that were conducted. 

### Cardiovascular risk factors 

The body mass index was used to assess obesity, calculated as the ratio of weight (kg) to height² (m). The results were then classified in accordance with the percentile curves for gender and age, as recommended by the Centers for Disease Control and Prevention,^18^ and as adapted for the Brazilian population through the Brazil Exports Project.^19^ After establishing percentile rates, body mass index values were classified in accordance with what has been described by the Centers for Disease Control and Prevention,18 as follows: low weight (P5); normal (≥ P5 and < P85); overweight (P ≥ 85 and < P95) and obese (≥ P95). From this, two groups were set up: group 1 (low weight and normal) and group 2 (overweight and obese). 

Abdominal obesity was also evaluated, using waist circumference measurements, with the individual standing in an upright position, with the hands hanging down close to the body. The waist measurements were made using a measuring tape marked in centimeters, using the reference point of the narrowest part of the trunk, between the ribs and the outer edge of the upper iliac bone. Students were defined as obese if their measurement was > 75th percentile, as established in the classification of Fernández et al.^20^

Blood pressure was checked with the student in a sitting position, at rest. For the brachial perimeter, a sphygmomanometer was used, with a stethoscope on the right arm. The blood pressure was classified according to the age, gender and height percentiles, and was considered to be normal when < 90th percentile, at the threshold between the 90th and 95th percentiles and hypertensive (stages 1 and 2) when > 95th percentile, as prescribed by the Brazilian Hypertension Guidelines.^21^

The biochemical variables (glucose, total cholesterol and triglycerides) were obtained through blood analysis, using the Accutrend GCT, and the results were then classified in accordance with the First Childhood and Adolescence Atherosclerosis Prevention Guidelines.^22^

The anthropometric, pressure and biochemical assessments were conducted by healthcare professionals with experience in assessments. Scholarship holder researchers were firstly trained so that they could help with data collection.

Data analysis was carried out using the Statistical Package for Social Sciences (SPSS) 18.0 software for Windows. Descriptive analysis was used for sample characterization, the chi-square test for categorical variables and Pearson's linear correlation for evaluating the relationship between obesity indicators and other cardiovascular risk factors. Differences were taken to be statistically significant when P ≤ 0.05. 

### RESULTS

From the analysis on the prevalence of cardiovascular risk factors (Table 1), it was observed that 11.8% of the boys were overweight and 12.7 were obese; among the girls, 13.1% were overweight and 11.9 were obese. With regard to the lipid and glycemic profile, the girls presented a significantly higher percentage of limiting and augmented triglycerides (55.9%) than shown by the boys (40.9%). On the other hand, the boys presented a significantly higher percentage of glucose intolerance (3.0%) than shown by the girls (0.3%). No significant difference between the genders was detected in relation to total cholesterol. With regard to high blood pressure, 6.8% of the boys were at the threshold or were hypertensive, for systolic blood pressure. For diastolic blood school environment, housing location, glycemia, total cholesterol pressure, the percentage of threshold or hypertensive students or diastolic blood pressure, when the groups of low and normal was significantly higher among the boys (12.2%).

**Table 1:** Demographic characteristics and prevalence of cardiovascular risk factors

Characteristics	Male - n (%)	Female - n (%)	P
**Age group**			
8 to 11 years (children)	114 (48.1)	132 (40.4)	0.041
12 to 17 years (adolescents)	123 (51.9)	195 (59.6)	
**School **			
Public	208 (87.8)	298 (91.1)	0.123
Private	29 (12.2)	29 (8.9)	
**Housing location **			
Downtown	29 (12.2)	29 (8.9)	0.191
Suburb	163 (68.8)	247 (75.5)	
Rural zone	45 (19.0)	51 (15.6)	
**BMI (body mass index) **			
Low weight	5 (2.1)	15 (4.7)	0.421
Normal	174 (73.4)	230 (70.3)	
Overweight	28 (11.8)	43 (13.1)	
Obesity	30 (12.7)	39 (11.9)	
**Waist circumference **			
≤ 75th percentile (normal)	192 (81.0)	265 (81.0)	0.539
> 75th percentile (abdominal obesity)	45 (19.0)	62 (19.0)	
**Glycemia **			
Normal	230 (97.0)	326 (99.7)	0.011
Glucose intolerance	7 (3.0)	1 (0.3)	
**Total cholesterol **			
Desirable	160 (67.5)	210 (64.2)	0.714
Threshold	52 (21.9)	80 (24.5)	
Abnormally high	25 (10.6)	37 (11.3)	
**Triglycerides**			
Desirable	140 (59.1)	144 (44.0)	< 0.001
Threshold	50 (21.1)	73 (22.3)	
Abnormally high	47 (19.8)	110 (33.6)	
**Systolic arterial pressure**			
Normotensive	221 (93.2)	305 (93.3)	0.887
Threshold	10 (4.2)	12 (3.7)	
Hypertensive	6 (2.6)	10 (3.1)	
**Diastolic arterial pressure **			
Normotensive	208 (87.8)	295 (90.2)	0.035
Threshold	27 (11.4)	22 (6.7)	
Hypertensive	2 (0.8)	10 (3.1)	

Regarding the classification of the anthropometric, pressure and biochemical variables among the students, in relation to their nutritional status (Table 2), it was observed that the overweight/obese children and adolescents presented significantly larger waist circumference and higher triglycerides and systolic blood pressure than shown by the children of low/normal weight. No significant differences were detected with regard to age group, school environment, housing location, glycemia, total cholesterol or diastolic blood pressure, when the groups of low and normal weight and overweight and obese individuals were compared.

**Table 2:** Classification of the anthropometric, biochemical and pressure variables according to the nutritional status of the schoolchildren

Characteristics	Male - n (%)		P	Female - n (%)		P
	Group 1	Group 2		Group 1	Group 2	
Age group						
8 to 11 years (children)	77 (43.8)	37 (60.7)	0.017	92 (37.4)	40 (49.4)	0.038
12 to 17 years (adolescents)	99 (56.2)	24 (39.3)		154 (62.6)	41 (50.6)	
School						
Public	155 (88.1)	53 (86.9)	0.482	221 (89.8)	77 (95.1)	0.110
Private	21 (11.9)	8 (13.1)		25 (10.2)	4 (4.9)	
Housing location						
Downtown	21 (12.0)	8 (13.1)	0.044	25 (10.2)	4 (4.9)	0.082
Suburb	115 (65.3)	48 (78.7)		188 (76.4)	59 (72.8)	
Rural zone	40 (22.7)	5 (8.2)		33 (13.4)	18 (22.2)	
Waist circumference						
≤ 75th percentile (normal)	174 (98.9)	18 (29.5)	< 0.001	238 (96.7)	27 (33.3)	< 0.001
> 75th percentile (abdominal obesity)	2 (1.1)	43 (70.5)		8 (3.3)	54 (66.7)	
Glycemia						
Normal	172 (97.7)	58 (95.1)	0.257	245 (99.6)	81 (100.0)	0.752
Glucose intolerance	4 (2.3)	3 (4.9)		1 (0.4)	0 (0)	
Total cholesterol						
Desirable	124 (70.4)	36 (59.0)	0.229	159 (64.6)	51 (63.0)	0.493
Threshold	36 (20.5)	16 (26.2)		62 (25.2)	18 (22.2)	
Abnormally high	16 (9.1)	9 (14.8)		25 (10.2)	12 (14.8)	
Triglycerides						
Desirable	112 (63.6)	28 (45.9)	0.020	121 (49.2)	23 (28.4)	0.005
Threshold	36 (20.5)	14 (23.0)		50 (20.3)	23 (28.4)	
Abnormally high	28 (15.9)	19 (31.1)		75 (30.5)	35 (43.2)	
Systolic arterial pressure						
Normotensive	170 (96.6)	51 (83.6)	0.002	234 (95.1)	71 (87.7)	0.021
Threshold	4 (2.3)	6 (9.8)		5 (2.1)	7 (8.6)	
Hypertensive	2 (1.1)	4 (6.6)		7 (2.8)	3 (3.7)	
Diastolic arterial pressure						
Normotensive	156 (88.6)	52 (85.2)	0.457	226 (91.9)	69 (85.2)	0.116
Threshold	18 (10.2)	9 (14.8)		15 (6.1)	7 (8.6)	
Hypertensive	2 (1.2)	0 (0)		5 (2.0)	5 (6.2)	

Group 1: low and normal weight; Group 2: overweight and obese.

Table 3 presents a correlation analysis between body mass index and waist circumference (dependent variables), and age, systolic and diastolic blood pressure (independent variables). It shows that there was a statistically significant difference with a weak correlation between these variables. No correlation was found between the total and abdominal obesity indicators and the biochemical variables.

**Table 3:** Correlation between body mass index (BMI) and waist circumference (WC) with independent variables

		Age	SBP	DBP	Glycemia	TC	TG
BMI	C	0.336*	0.415*	0.376*	0.117	0.057	0.217
	P	< 0.001	< 0.001	< 0.001	0.006	0.188	< 0.001
WC	C	0.386*	0.461*	0.433*	0.143	0.043	0.199
	P	< 0.001	< 0.001	< 0.001	0.001	0.325	< 0.001

C = Pearson's linear correlation

P = significance level (P < 0.05)

* Weak correlation (r = 0.30 to r = 0.49)

SBP = systolic blood pressure

DBP = diastolic blood pressure

TC = total cholesterol

TG = triglycerides

### DISCUSSION

The assessment on the presence of cardiovascular risk factors showed that the prevalences of overweight and obesity among the boys were 11.8% and 12.7%, while they were 13.1% and 11.9%, respectively, among the girls. There was no significant differences between the genders. A study conducted among children in Greece detected higher potential for obesity and overweight, which together amounted to 42.1% among the boys and 39.8% among the girls.^23^ Higher obesity and overweight prevalence was respectively described in the Aden Governorate of Yemen^24^ (12.7% and 8.0%), China^25^ (11.5% and 10.3%), Spain^26^ (18.8% and 10.3%) and the United States^27^ (16.0% and 18.0%). In Germany,^28^ 14.5% were found to present excessive weight.

Among children and adolescents in Paraná, Brazil, results similar to those of the present study were detected, such that 28.0% of the boys and 24.7% of the girls presented conditions of obesity/overweight.^29^ In Minas Gerais, the prevalence of overweight/obesity reached 13.8% among the boys and 9.7% among the girls.^30^ Also in Brazil, Mazaro et al.^31^ evaluated children aged 7 to 11, in Sorocaba, state of São Paulo, and found that 22.05% presented excessive weight (13.08% overweight and 8.97% obese). In the state of Pernambuco,^32^ excessive weight conditions affected 11.6% and 15.0% of the children and adolescents, respectively. In comparative terms, our findings exceeded the prevalence of previous studies, such that excessive weight conditions affected 31.3% of the children and 20.4% of the adolescents. A study conducted in Campinas, state of São Paulo, demonstrated that obesity was occurring rather early in childhood, given that babies with BMI greater than the 97th percentile were identified.^33^ In Taubaté, state of São Paulo, two to three-year-old children also showed high prevalence of overweight conditions (28.86%).^34^

In addition to the high prevalence of total obesity, 19.0% of the boys and girls suffered from abdominal obesity. In India, the prevalence of abdominal obesity among schoolchildren reached 4.5%.^35^ In Portuguese children, this prevalence amounted to 21.3%.^36^ In Ecuadorian children, the abdominal obesity rates reached (19.7%), i.e. similar to what was found in the present study.^37^ A study comparing two Brazilian regions showed prevalences of abdominal obesity of 8.9% and 4.2% among boys and girls, respectively, in western Santa Catarina, and 3.5% (boys) and 1.1% (girls) in northern Minas Gerais.^38^ In Londrina, Paraná, the overall abdominal obesity prevalence among all the adolescents evaluated was 15.2% among boys and 8.5% among girls.^39^

The vast majority of the schoolchildren in the overweight/ obese group presented lipid profile alterations. With regard to total cholesterol, the boys showed a greater difference between the groups, such that 41.0% of the overweight/obese schoolchildren presented altered total cholesterol levels. Regarding triglycerides, altered results were found particularly among the overweight/obese girls (71.6%). Although the overweight/obese schoolchildren showed higher results for the biochemical variables, compared with the normal/low weight schoolchildren, no correlation was found for body mass index and waist circumference in relation to total cholesterol, triglycerides and glycemia. A study carried out in Florianópolis among children and adolescents demonstrated that excess weight was the most serious risk factor associated with dyslipidemia.^40^ In Natal, Rio Grande do Norte, a study conducted among children and adolescents concluded that altered total cholesterol levels were especially prevalent in the male overweight/obese groups, thus attesting to the accuracy of the present study. Regarding triglycerides, most alterations were particularly found among the obese girls.^41^

Among schoolchildren evaluated in Itajaí, Santa Catarina, it was demonstrated that for the lipid profile evaluated, only highdensity lipoprotein (HDL) was associated with the presence of obesity.^42^ Xu et al.^25^ evaluated Chinese schoolchildren aged 7 to 11 and concluded that the prevalences of high triglycerides, low HDL-cholesterol, hypertension and high glucose among children with normal weight were all significantly lower than those among overweight and obese children. A study conducted in São Paulo showed high prevalence of insulin and impaired glucose tolerance relating to trunk and body fat among obese nondiabetic adolescents.^43^

The present study also found that the overweight/obese schoolchildren presented higher altered systolic and diastolic blood pressure. Consequently, in the overweight/obese group, 16.4% of the boys and 12.3% of the girls presented altered systolic blood pressure and 14.8% of the boys and 14.8% of the girls showed altered diastolic blood pressure. A weak correlation was found between the abdominal/total obesity indicators and the systolic and diastolic blood pressure. In a study conducted among schoolchildren in Santa Cruz do Sul, Rio Grande do Sul, and published two years ago, Burgos et al.^15^ concluded that a growing number of children and adolescents are reaching the threshold, or are hypertensive, proportionally to increases in the body mass index classification. These authors also detected a weak correlation between body mass index and blood pressure (both systolic and diastolic). In Salvador, the high percentage of altered systolic and diastolic blood pressure kept pace with higher body mass index, with a 46.4% incidence among the boys and 39.3% among the girls with obesity, for systolic blood pressure, and 42.0% among the obese boys and 44.6% among the obese girls, for diastolic blood pressure.^44^ In Mexico City, considerably higher values were detected among obese schoolchildren than among normal weight children, only for systolic blood pressure. For diastolic blood pressure, no significant differences were detected between the two groups.^45^ In Germany, overweight and obese children and adolescents presented significantly higher values, both for systolic blood pressure and for diastolic blood pressure, compared with normal weight schoolchildren.^46^

Abdominal obesity, measured in terms of the waist circumference, also showed weak but significant correlations with systolicblood pressure (r = 0.461) and diastolic blood pressure (r = 0.433) in the present study. Similar results were found in an Italian study, which also shows weak but significant correlations between waist circumference and systolic blood pressure (r = 0.40) and diastolic blood pressure (r = 0.29).^47^ In Madrid, Spain, adolescents presented statistically significant differences in abdominal obesity in relation to systolic blood pressure and average blood pressure.^13^ In schoolchildren in Buenos Aires, an association between abdominal obesity and systolic and diastolic blood pressure was also detected. That study also pointed towards waist circumference as a predictor of resistance to insulin, thus suggesting that this test could be included in clinical practice, since it is a very easy tool for identifying children at risk of developing metabolic syndrome.^48^

Childhood obesity tends to continue throughout adult life, mainly due to inappropriate habits relating to sedentarism and poor diet that are acquired early in life and tend to persist throughout adulthood. Furthermore, excess weight during childhood and adolescence years may entail irreversible alterations to hormonal clusters, adipose cells and even the brain, thereby leading to an increased sense of hunger and negatively affecting metabolic functions.^49^ Physical exercises are extremely important in treating obese children and adolescents, since they boost the arterial blood flow, thus primarily acting towards prevention of atherosclerosis.^50^

Because of the different patterns among the Brazilian population, with regard to its demographic, social and economic heterogeneity, identification of the prevalence of overweight/obesity and other cardiovascular disease factors not only helps in understanding the related environmental factors, but also contributes towards knowledge of the reality of the population in question.^51^ This makes it possible to evaluate the demands and aids in drawing up public health strategies focused on disease prevention and health promotion.^52^ In this manner, it will become possible for all public administrators to develop specific initiatives, in accordance with every region's needs.

Therefore, prevention of childhood obesity is the key to reducing the obesity rates among adults and reducing the comorbidity and mortality rates relating to this epidemic, which has been growing drastically, in both developed and developing countries.^53^ This situation highlights the importance of identifying and monitoring these cardiovascular risk factors from childhood onwards. Additionally, through engaging different professionals within healthcare, school and public entities, and equally involving the parents of the schoolchildren, it will be possible to develop and set up prevention strategies. This can particularly be achieved through changes to children and adolescents' lifestyles, such that habits that are more active are promoted, with healthier diets. Thus, through improving the lifestyles of these children and adolescents, such that they acquire healthy habits from the very beginning, the risk of developing chronic-degenerative diseases in adulthood will decrease significantly, while the life expectancy of the population increases.

### CONCLUSION

There is a high prevalence of both overweight and obese conditions in both genders among children in Santa Cruz do Sul. Moreover, overweight/obese schoolchildren presented much higher percentages of cardiovascular risk factors than shown by low/normal weight schoolchildren. A weak but significant correlation was detected between the total/abdominal indicators and systolic and diastolic blood pressure.
